# Barriers to Access to Treatment for Hypertensive Patients in Primary Health Care of Less Developed Northwest China: A Predictive Nomogram

**DOI:** 10.1155/2021/6613231

**Published:** 2021-04-14

**Authors:** Lin Wang, Mulalibieke Heizhati, Xintian Cai, Mei Li, Zhikang Yang, Zhongrong Wang, Reyila Abudereyimu, Nanfang Li

**Affiliations:** ^1^Hypertension Center of People's Hospital of Xinjiang Uygur Autonomous Region, Xinjiang Hypertension Institute, National Health Committee Key Laboratory of Hypertension Clinical Research, Urumqi, Xinjiang, China; ^2^Xinjiang Medical University, Urumqi, China

## Abstract

**Background:**

This study aims to evaluate the risk factors associated with untreated hypertension and develop and internally validate untreated risk nomograms in patients with hypertension among primary health care of less developed Northwest China.

**Methods:**

A total of 895 eligible patients with hypertension in primary health care of less developed Northwest China were divided into a training set (*n* = 626) and a validation set (*n* = 269). Untreated hypertension was defined as not taking antihypertensive medication during the past two weeks. Using least absolute shrinkage and selection operator (LASSO) regression model, we identified the optimized risk factors of nontreatment, followed by establishment of a prediction nomogram. The discriminative ability, calibration, and clinical usefulness were determined using the area under the receiver operating characteristic curve (AUC), calibration curve, and decision analysis. The results were assessed by internal validation in the validation set.

**Results:**

Five independent risk factors were derived from LASSO regression model and entered into the nomogram: age, herdsman, family income per member, altitude of habitation, and comorbidity. The nomogram displayed a robust discrimination with an AUC of 0.859 (95% confidence interval: 0.812–0.906) and good calibration. The nomogram was clinically useful when the intervention was decided at the untreated possibility threshold of 7% to 91% in the decision curve analysis. Results were confirmed by internal validation.

**Conclusions:**

Our nomogram showed favorable predictive accuracy for untreated hypertension in primary health care of less developed Northwest China and might help primary health care assess the risk of nontreatment in patients with hypertension.

## 1. Introduction

Hypertension is the leading risk factor for cardiovascular disease (CVD), which remains an important public health problem as one of the top causes of death in China, causing heavy social, familial, and economic dysfunctions [1, 2]. The prevalence of hypertension is about 27.9% in the population aged ≥18 years [3] and up to 41.9% in the population aged ≥35 years in China [4], but the hypertension treatment rate is only 40.7%, which is even unacceptably low (22.1%) in underdeveloped northwest China compared with southeast of China (51.4%) [5, 6].

For hypertensive patients, antihypertension treatment is fundamental to improve health outcomes, minimize their impact, prevent further disability, and reduce health care costs [7, 8]. Despite the fact that hypertension is one of the most common chronic conditions treated in primary care [9] and the fact that the benefit of reducing blood pressure (BP) levels in hypertensive patients has been demonstrated [10, 11], it is untreated in a significant proportion of patients, even those who are aware that they have hypertension. Compared with European and American countries, the treatment rate of hypertension is increasing rather slowly in China owing to the unique social medical system, health care policy, family economic restrictions, traffic obstacle, and so forth [12–15]. Therefore, there remain enormous challenges in the understanding and primary prevention of hypertension in China.

Northwest China is economically less developed, with large land area and sparse population density. One-fifth of local residents are leading nomadic or seminomadic lives, making the penetration rate of medical resources low, health awareness poor, and the lifestyle unhealthy [16, 17]. All these factors may lead to higher prevalence of hypertension and its poor treatment especially among residents in stock-raising regions. However, antihypertensive medication nontreatment is affected by multiple determinants such as patient-related factors (e.g., age, sex, education level, and access to health care), socioeconomic factors (e.g., occupation, marital status, emotional support, and family income), and condition-related factors (e.g., health status and comorbidity) [18].

Considering so many associated risk factors, accurate prediction nontreatment tools and early intervention may be the most effective actions toward unsatisfactory treatment. Therefore, this study aimed to identify the risk factors associated with untreated hypertension in the management of hypertension in primary health care of Northwest China and to develop a predictive nomogram to estimate the probability of nontreatment in a given visit, according to five dimensions related to the demographic factors, socioeconomic status, living environment, health-related behaviors, and anthropometric value.

## 2. Materials and Methods

### 2.1. Study Design and Population

The cross-sectional study is reported according to the STROBE checklist standards. This study design and methods have been described previously [19]. Briefly, we used four-stage (city/county-district/township-community/village-resident) stratified random sampling method to obtain the study samples aged ≥15 years across Xinjiang, Northwest China, between Augusts 2014 and September 2015. Totally there are 84 sites (60 villages and 24 communities) in 5 rural counties and 2 urban districts including 7276 participants (with response rate >95%) in the survey. The inclusion criteria were as follows:

1. Residents who are willing to participate in the investigation and sign an informed consent form
2. Local inhabitants aged ≥15 years
3. Residing at the current address for ≥6 months
4. Women who are not pregnant

**Figure 1:**
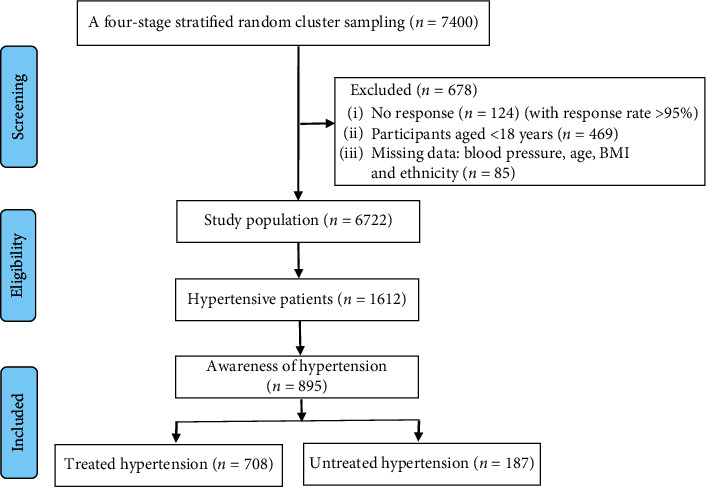
The flow chart of inclusion and screening of surveyed subjects.

For the current analysis, we excluded participants without response (*n* = 124) and participants aged <18 years (*n* = 469). We selected 1,612 hypertensive patients and further excluded patients who were unaware that they have hypertension (*n* = 717), since patients who were unaware of having hypertension would not be on antihypertensive treatment at the time of survey. Therefore, 895 subjects who were confirmed to be diagnosed with hypertension before the survey of this study were included in the final analysis as shown in Figure 1. The Ethics Committee of People's Hospital of Xinjiang Uygur Autonomous Region and the Fuwai Hospital Ethics Review Board approved the current study.

### 2.2. Data Collection and Measurement

Trained study staff used a standardized questionnaire to collect data on demographic characteristics (such as sex, age, and ethnicity), socioeconomic status (occupation, education attainment status, marital status, and family income per member), health-related behaviors (alcohol consumption and cigarette smoking) [19], and hypertensive status (whether it was previously diagnosed by a doctor, whether it has been treated, and whether taking antihypertensive drugs within the previous two weeks).

### 2.3. Anthropometric Variables

Trained observers measured the body height, weight, waist circumference (WC), and blood pressure (BP) of each participant according to the standardized equipment and procedures. In order to protect the privacy of participants during the anthropometric measurements, we arranged the measurement site in a room with a suitable temperature, and only one participant can enter at a time. In addition, participants wore light clothing, while their weight and waist circumference were measured. Each participant's BP records were measured by using the automatic sphygmomanometer OMRON HBP-1300 Professional Portable Blood Pressure Monitor (OMRON, Kyoto, Japan) three times on the right upper arm after the participant rested 5 min in a seated position, with 30 seconds between each measurement with an observer present. The mean value of the three measurements was used for analysis. Body mass index (BMI) was calculated as weight divided by the square of height (kg/m^2^).

### 2.4. Plasma Glucose and Lipid Measurements

All subjects fasted for ≥8 h, and a 5 mL fasting blood sample was collected. Next, fasting plasma glucose (FPG), triglyceride (TG), total cholesterol, low-density lipoprotein cholesterol (LDL-C), and high-density lipoprotein cholesterol (HDL-C) were tested using standard methods.

### 2.5. Definitions

*Hypertension* was defined as systolic blood pressure ≥140 mm Hg and/or diastolic blood pressure ≥90 mm Hg and/or use of antihypertensive medicine within 2 weeks and/or self-reported presence of hypertension [20]. *Untreated hypertension* was defined as not taking antihypertensive medication during the past two weeks. *Occupational status* was defined as herdsman and nonherdsman. *Education attainment status* was categorized into three levels: primary and lower, junior high, and senior high and higher. *Marital status* was coded as single, married, or separated. *Family income per member* was categorized into four levels: <￥500/month, ￥500–1000/month, ￥1001–3000/month, and >￥3000/month. *Current smokers* were defined as participants who have smoked at least 20 packets of cigarettes in their lifetime and are currently smoking cigarettes or smokeless tobacco [19]. *Current drinkers* were defined as participants who consumed at least once alcoholic beverage per week in the past month. The BMI was further classified into *normal, overweight, and obesity* if BMI is below 24.0 kg/m^2^, between 24.0 and 27.9 kg/m^2^, and ≥28.0 Kg/m^2^, respectively. *Abdominal obesity* was defined as WC ≥ 90 cm in men and WC ≥ 85 cm in women. *Diabetes* was defined as FPG ≥ 7.0 mmol/L or self-reported presence of diabetes. *Dyslipidemia* was defined as a combination of one or more statuses: TC ≥ 6.22 mmol/L, LDL-C ≥  4.14 mmol/L, HDL-C < 1.04 mmol/L, TG ≥ 2.26 mmol/L, and self-reported presence of dyslipidemia in terms of criteria recommended by Chinese guidelines for the prevention and treatment of dyslipidemia in adults [21]. *Comorbidity* was defined as a combination of one or more diseases: CVD (coronary heart disease, stroke), diabetes, and dyslipidemia.

### 2.6. Statistical Analysis

Descriptive data were conducted for 895 subjects at baseline using SPSS 20.0 for Windows (SPSS Inc., Chicago, IL). Continuous variables were presented as means ± standard deviations (M ± SD) and categorical variables were expressed as frequency (*n*) and proportion (%), and the results were compared using Student's *t*-test, the chi-square test, and Mann-Whitney *U* test to detect the statistical significance, respectively.

Steps of the formulation and assessment of the nomogram were carried out using the package of “rms” in R version 3.5.1 (http://www.r-project.org/) as follows: First, we extracted the training set derived from the 70% (*n* = 626) of randomized samples of the total sample population for use in building the nomogram. We reserved the remaining 30% (*n* = 269) as validation set for the validation. The least absolute shrinkage and selection operator (LASSO) method was used to identify independent predictive features from the training set by nonzero coefficients in the LASSO regression model [22–24]. Second, we used multivariable logistic regression analysis to build a predicted nomogram based on the selected features from the LASSO regression model [25, 26], with results presented as *β* and odds ratio (OR) with associated 95% confidence interval (95% CI). Third, the discrimination and calibration of the nomogram were assessed by the area under the receiver operating characteristic curve (AUC) and calibration curves plot, respectively [27]. In addition, the nomogram accuracies were evaluated by internal validation, in which we calculated an AUC in the validation set. Finally, to quantify the net benefits at different threshold probabilities in the study, decision curve analysis was conducted in the validation set to determine the usefulness of the nontreatment nomogram [28]. All statistical tests were two-sided, and *P* values < 0.05 were considered to be statistically significant.

## 3. Results

### 3.1. Patient Characteristics

In total, 895 patients with mean age of 52.65 ± 17.49 years were enrolled, with women accounting for 52.7%. The baseline characteristics between training and validation sets are displayed in Table 1. No statistically significant differences in age, sex, education level, ethnicity, number of family members, marital status, altitude of habitation, current smokers, current drinkers, BMI, abdominal obesity, and comorbidity were observed between the two sets.

Table S1 showed that the untreated rate among patients with hypertension was 20.9% (187/895). As compared with patients under antihypertensive treatment, those who were untreated were more likely to be older, be herdsmen, experience lower education status, and have less family income per member and less likely to have comorbidity.

### 3.2. Selected Factors for Model

**Figure 2:**
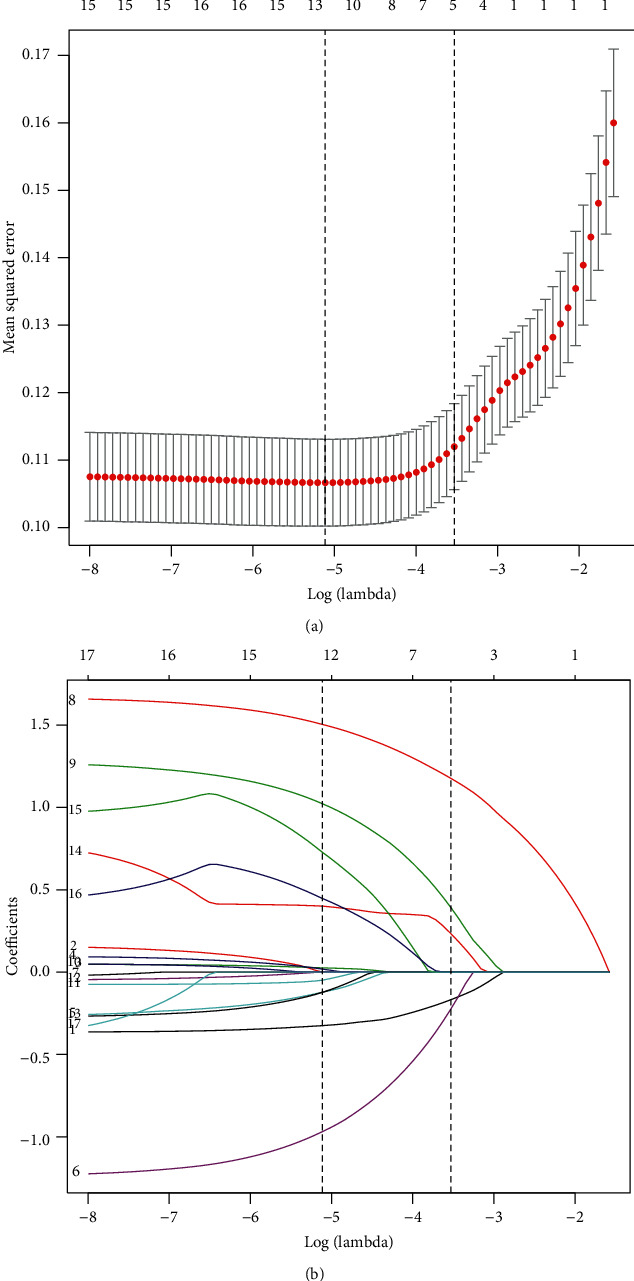
Demographic features, socioeconomic status, live setting, health-related behaviors, and anthropometric value selection using the LASSO binary logistic regression model. (a) Optimal parameter (lambda) selection in the LASSO model used fivefold cross-validation via minimum criteria. The partial likelihood deviance (binomial deviance) curve was plotted versus log (lambda). Dotted vertical lines were drawn at the optimal values by using the minimum criteria and the 1-SE of the minimum criteria (the 1-SE criteria). (b) LASSO coefficient profiles of the 17 features. A coefficient profile plot was produced against the log (lambda) sequence. Vertical line was drawn at the value selected using fivefold cross-validation, where optimal lambda resulted in five features with nonzero coefficients. LASSO: least absolute shrinkage and selection operator; SE: standard error.

The samples of the training set were used for building models. Of demographic features, socioeconomic status, living region, health-related behaviors, and anthropometric values, 17 factors were reduced to five potential predictors in the study (∼3 : 1 ratio; Figures 2(a) and 2(b)) and were with nonzero coefficients in the LASSO regression model. These factors included age, herdsman, family income per member, altitude of habitation, and comorbidity (Table 2).

### 3.3. Development of Predictive Nomogram

**Figure 3:**
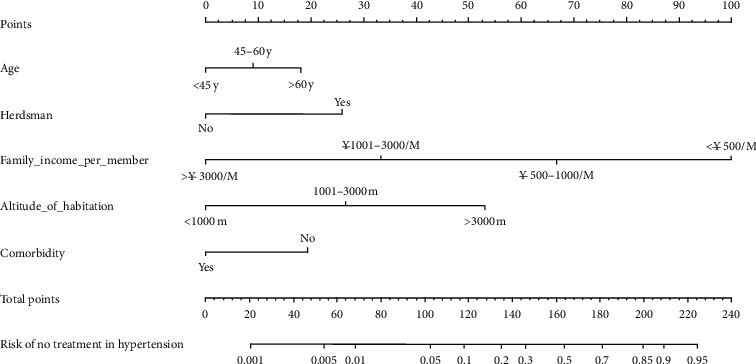
Developed medication nontreatment nomogram.

The results of multivariable logistic regression analysis were shown in Table 2, which included the above independent predictors. Then, the model was built and presented as the nomogram (Figure 3). We observed that family income per member <￥500/month was corresponding to the highest risk score of 100 points, and second was the altitude of habitation >3000 m (53 points), followed by herdsman (26 points), no comorbidity (19 points), and age > 60 years (18 points).

### 3.4. Discriminative Ability, Validation, and Calibration of the Nomogram

**Figure 4:**
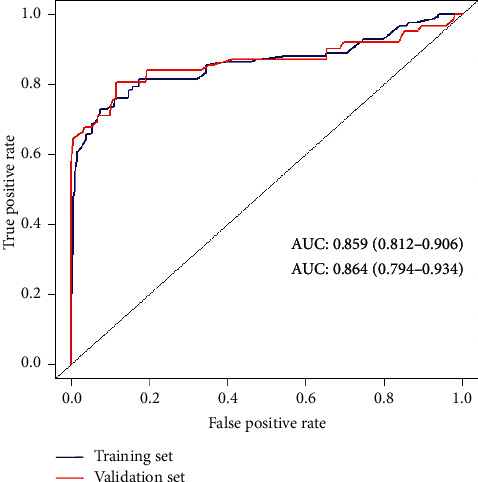
Receiver operating characteristic curve of the untreated nomogram prediction in the study.

**Figure 5:**
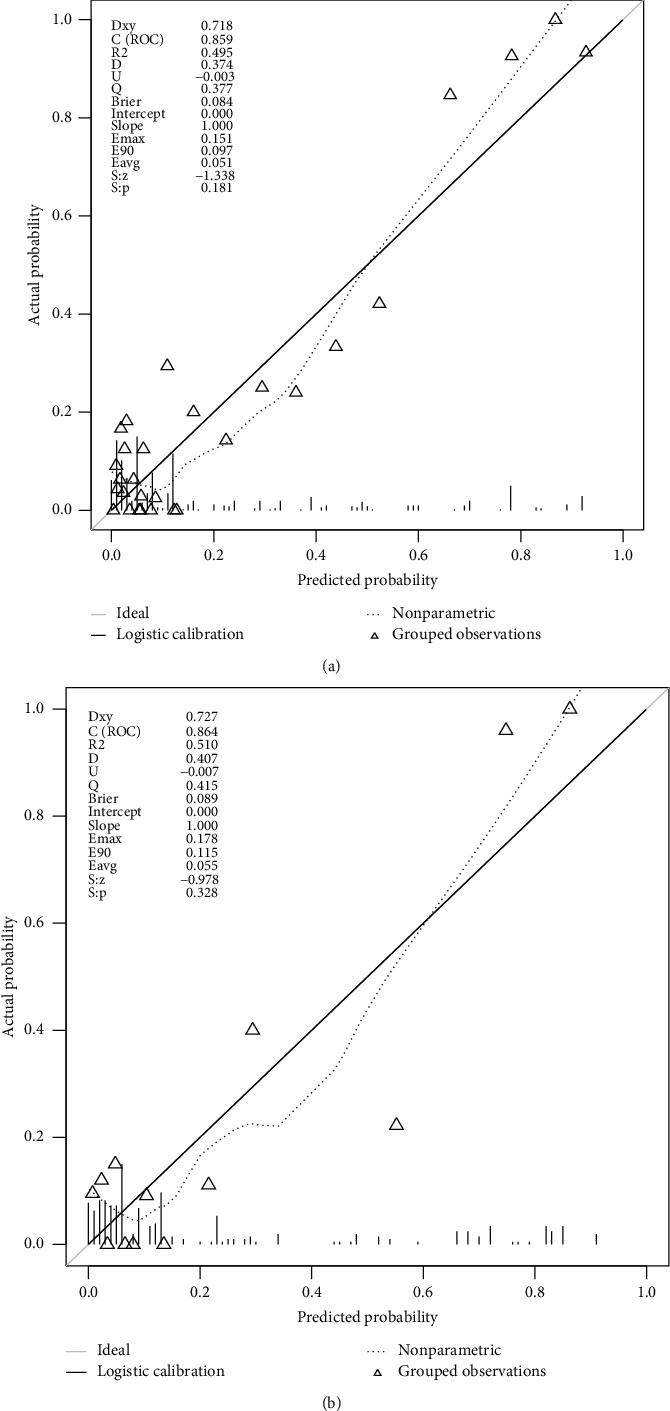
Calibration curves of the nomogram in the training set (a) and validation set (b). The *x*-axis represents the predicted untreated risk. The *y*-axis represents the actual identified untreated patients. The diagonal dotted line represents a perfect prediction by an ideal model. The solid line represents the performance of the nomogram, of which a closer fit to the diagonal dotted line represents a better prediction.

The AUC for the prediction nomogram was 0.859 (95% CI: 0.812–0.906) (Figure 4), and it was confirmed to be 0.864 (95% CI: 0.794–0.934) through internal validation, indicating the model's good discrimination. Calibration of the untreated risk nomogram for the prediction of untreated risk in hypertensive patients was performed by the calibration curve plot. When *P* value > 0.05, the calibration ability of the model is good. The calibration curve of the training set was *P*=0.181, and the calibration curve of the validation set was *P*=0.328, all of which were greater than 0.05, demonstrating that the model had good calibration ability (Figures 5(a) and 5(b)).

### 3.5. Clinical Use

**Figure 6:**
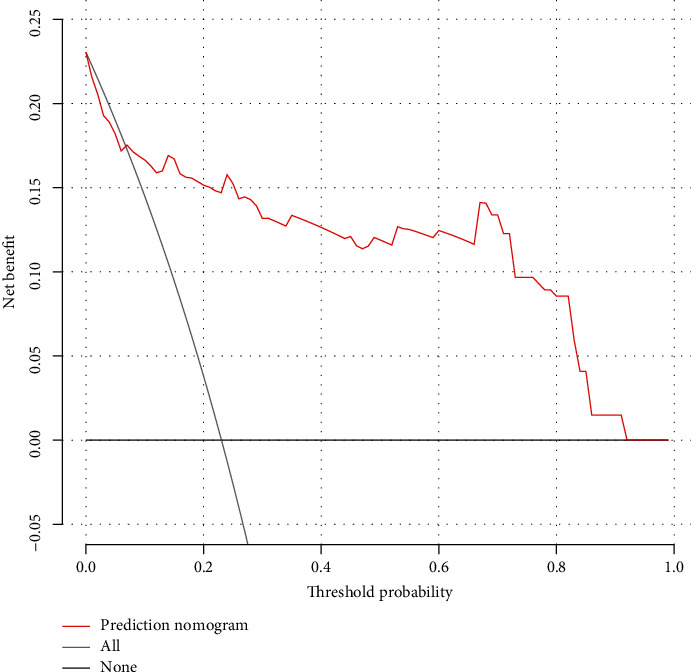
Decision curve analysis for the untreated nomogram. The *y*-axis measures the net benefit. The red line represents the medication untreated risk nomogram. The thin solid line represents the assumption that all patients are nontreatment to medication. Thin thick solid line represents the assumption that no patients are nontreatment to medication. The decision curve showed that if the threshold probability of a patient ranges from 7% to 91%, using this untreated nomogram in the current study to predict medication nontreatment risk adds more benefit than the intervention-all-patients scheme or the intervention-none scheme.

The decision curve analysis for the untreated nomogram showed that if the threshold probability of a hypertensive patient ranges from 7% to 91%, using this untreated nomogram to predict untreated risk adds more benefit than the scheme (Figure 6).

## 4. Discussion

Herein, this is the first study in relatively representative patients with hypertension among primary health care of less developed northwest China to develop a predictive nomogram to evaluate the factors influencing the access to treatment for hypertension. Untreated rate of hypertension is still very high among patients with hypertension, despite the existence of universal access and the availability of effective treatments. The nomogram developed is simple (consisting of only five factors; during selection of variables for each block, many were eliminated because they were not associated with nontreatment or because they were strongly collinear with other variables) and shows good standardization and ability to discriminate. Its high sensitivity (85%) is worth mentioning, indicating that the factors included are able, as a whole, to predict properly hypertensive patients who have no access to treatment.

Herdsmen are those who are leading a nomadic or seminomadic lifestyle. Common antihypertension agents are not readily available in many stock-raising regions due to their nomadic lifestyles. In addition, the proportions of individuals who could not afford these drugs are much higher among them, based on their household income [29, 30]. It has also been indicated that hypertensive individuals often stop taking antihypertensive drugs when BP control is achieved [31]. Similar to previous studies, our findings suggested that family income per member <￥500/month was also associated with higher nontreatment. Furthermore, the use of antihypertensive drug treatment patterns by village physicians is different compared with tertiary hospital physicians [31]. Village doctors in stock-raising regions may lack knowledge or willingness to follow new guidelines and might have been entrenched in traditional prescription habits due to obstacles in information exchange [4]. Patients often give up on continuing antihypertensive treatment for suboptimal pharmacotherapy [32]. Therefore, it may explain, at least in part, why untreated rates of hypertension are high.

With the increase in geographical altitude, the population will become sparse, the style of living and production will become more singular, and health needs and access to health care become considerable challenges in high-altitude regions [33, 34]. The presence of hypertension is common in residents living in high-altitude regions [35, 36] and requires pharmacological therapy in the majority of cases in order to manage them properly. Some studies show that the prevalence of polypharmacy and primary health care is associated with the prevalence of chronic diseases [37, 38]. To our surprise, the factor of altitude of habitation is a key point to affect treatment. High altitude may result in poor treatment due to the absence of an adequate health network in high-altitude areas able to properly provide care and necessary BP-lowering agents to the residents [39], limiting the consequences in their antihypertensive performance. It has been reported that about 83 million people reside in highlanders (defined as >2,500 meters above sea level) [40], distributed mainly in South America, Eastern Africa, and Central Asia [41]. The prevalence of hypertension at high-altitude locations is as high as 55.9% [42]. Therefore, given the large number of population from highlanders, the huge burden of hypertension there and its well-established role in cardiovascular risk, further increasing the density of primary care providers or services and the access to medicines for hypertension in high-altitude areas, especially in regions above 3000 m, may enhance treatment in hypertensive patients.

The elderly and hypertensive patients without comorbidities were associated with nontreatment. This may be explained by the fact that elderly people in rural and stock-raising regions have traffic obstacles when they go to town to buy antihypertensive drugs and most elderly farmers or herdsmen generally lose a large part of their economic resources compared with their younger counterparts, which may have led them to take antihypertensive drugs intermittently [31]. Hypertensive patients without comorbidity may be paying less attention to their health condition compared with those with comorbidity, which may also contribute to these patients without comorbidity being a predictor for untreated hypertension. Accordingly, increasing access to health care and strengthening health education and regular follow-up target adherence may be vital ways to further improve hypertension treatment [43, 44].

Our study has several strengths. First, the nontreatment risk prediction tools may provide important insight to clinicians in delivering optimal health care services. Using accessible metrics such as age, occupation, number of family members, altitude of habitation, and information about comorbidity, this tool can help the clinicians, especially primary health providers, to better identify patients who are at high risk of not taking medications as prescribed. Finding such patients is an important step before intervening to improve their adherence. Second, our model showed good accuracy and excellent agreement in training set and validation set, which suggests that it has good transportability and generalizability.

Some limitations in the current report should be kept in mind when interpreting the results. First, the study sample may not stand for all Chinese with hypertension. Patients who are not aware of their hypertensive status were also excluded from the analysis. Second, risk factor analysis may have missed some potential variables that could affect the commence of antihypertensive treatment such as the therapy-related factors (e.g., type of medicine, side effects, and medicine-related questions) and social support. Third, although the robustness of our nomogram was examined with internal validation, we failed to perform external validation, and therefore the generalizability of current data may be limited in other populations and regions/countries and relevant external validation is needed.

## 5. Conclusions

There is a considerable nontreatment rate among hypertensive patients in primary health care of less developed Northwest China. This study developed a novel nomogram with a relatively good accuracy to help primary health care access the risk of nontreatment in hypertensive patients when they manage these patients. Herdsmen living in high-altitude areas with low family income may be the key population for enhancing antihypertensive treatment.

## Figures and Tables

**Table 1:** Baseline characteristics of the study population by training set and validation set.

Variables	Training set (*n* = 626)	Validation set (*n* = 269)	Total (*n* = 895)	*P* value
Age (years)	52.63 ± 17.61	52.69 ± 17.26	52.65 ± 17.49	0.963 ^*∗*^
<45 y	191 (30.5)	77 (28.6)	268 (29.9)	0.820^‡^
45–60 y	201 (32.1)	91 (33.8)	292 (32.6)	
>60 y	234 (37.4)	101 (37.5)	335 (37.5)	

Gender, women (*n*,%)	337 (53.8)	135 (50.2)	472 (52.7)	0.316^#^
Herdsman (*n*, %)	260 (41.5)	116 (43.1)	376 (42.0)	0.659^#^

Education levels (*n*, %)				
Primary and lower	361 (57.7)	159 (59.1)	520 (58.1)	0.923^‡^
Junior high	145 (23.2)	60 ( 22.3)	205 (22.9)	
Senior high and higher	120 (19.2)	50 (18.6)	170(19.0)	

Ethnicity (*n*, %)				
Han	269 (43.0)	111 (41.3)	380 (42.5)	0.198^#^
Kazakh	90 (14.4)	54 (20.1)	144 (16.1)	
Tajik	74 (11.8)	28 (10.4)	102 (11.4)	
Others	193 (30.8)	76 (28.3)	269 (30.1)	

Number of family members (*n*, %)				
1	33 (5.3)	10 (3.7)	43 (4.8)	0.551^‡^
2–4	403 (64.4)	172 (63.9)	575 (64.3)	
≥5	190 (30.4)	87 (32.3)	277 (30.9)	

Marital status (*n*, %)				
Single	64 ( 10.2)	21 (7.8)	85 (9.5)	0.508^#^
Married	511 (81.6)	224 (83.3)	735 (82.1)	
Separated	51 (8.1)	24 (8.9)	75 (8.4)	

Family income per member				
<￥500/month	142 (22.7)	65 (24.2)	207 (23.1)	0.004^‡^
￥500–1000/month	73 (11.7)	30 (11.2)	103 (11.5)	
￥1001–3000/month	286 (45.7)	146 (54.3)	432 (48.3)	
>￥3000/month	125 (20.0)	28 (10.4)	153 (17.1)	

Altitude of habitation (m)				
<1000	387 (61.8)	164 (61.0)	551 (61.6)	0.717^‡^
1000–3000	163 (26.0)	76 (28.3)	239 (26.7)	
>3000	76 (12.1)	29 (10.8)	105 (11.7)	

Current smokers (*n*, %)	150 (24.0)	50 (18.6)	200 (22.3)	0.077^#^
Current drinkers (*n*, %)	138 (22.0)	46 (17.1)	184 (20.6)	0.093^#^

Body mass index	27.29 ± 4.30	27.25 ± 4.48	27.28 ± 4.35	0.917 ^*∗*^
BMI: <23.9 kg/m^2^	124 (19.8)	53 (19.7)	177 (19.8)	0.669^‡^
BMI: 24.0–27.9 kg/m^2^	242 (38.7)	112 (41.6)	354 (39.6)	
BMI: ≥28.0 kg/m^2^	260 (41.5)	104 (38.7)	364 (40.7)	

Abdominal obesity (n, %)	424 (67.7)	183 (68.0)	607 (67.8%)	0.930^#^
CVD (*n*, %)	21 (3.4)	2 (0.7)	23 (2.6)	0.195^#^
Diabetes (*n*, %)	91 (14.5)	31 (11.5)	122 (13.6)	0.228^#^
Dyslipidemia (*n*, %)	113 (18.1)	41 (15.2)	154 (17.2)	0.307^#^
Comorbidity (*n*, %)	193 (30.8)	66 (24.5)	259 (28.9)	0.057^#^

Blood pressure (mmHg)				
Systolic blood pressure	149.37 ± 20.58	152.09 ± 21.34	150.19 ± 20.83	0.073 ^*∗*^
Diastolic blood pressure	85.67 ± 12.31	85.97 ± 13.68	85.76 ± 12.73	0.749 ^*∗*^

CVD, cardiovascular disease.  ^*∗*^Student's *t*-test for continuous variables. ^‡^Mann-Whitney *U* test for ordered multicategorical variables. ^#^Chi-square test for binary variables.

**Table 2:** Prediction factors for nontreatment in hypertension from study population by multiple logistic regression model.

Stratification	*β*	OR (95% CI)	*P* value
Age	0.517	1.68 (1.27–2.21)	<0.001
Herdsman	1.054	2.87 (1.77–4.64)	<0.001
Family income per member	−1.644	0.19 (0.15–0.25)	<0.001
Altitude of habitation	1.134	3.11 (2.17–4.44)	<0.001
Comorbidity	−0.776	0.46 (0.27–0.78)	0.004
Constant	−1.920	0.147	<0.001

OR: odds ratio; CI: confidence interval.

## Data Availability

Materials included in the manuscript, excluding the relevant raw data, will be made freely available to any researchers who wish to use them for noncommercial purposes, while preserving any necessary confidentiality and anonymity.

## Supplementary Materials

Supplementary Materials Table S1: Baseline characteristics of the treatment and nontreatment groups of the study population.
