# Supplementary material for: Barriers to Access to Treatment for Hypertensive Patients in Primary Health Care of Less Developed Northwest China: A Predictive Nomogram
Source: Int J Hypertens. 2021 Apr 14;2021:6613231. doi: 10.1155/2021/6613231 (PMC8062209; doi:10.1155/2021/6613231)
Supplement: Supplementary Materials — Table S1: Baseline characteristics of the treatment and nontreatment groups of the study population. [file 6613231.f1.docx]

| Supplementary Table 1: Baseline characteristics of the study population by treatment and non-treatment groups | | | | |
| --- | --- | --- | --- | --- |
| Variables | Treatment (n=708) | Non-treatment (n=187) | Total (n=895) | P value |
| Age (years) | 50.81±17.96 | 59.64±13.56 | 52.65±17.49 | <0.001 |
| <45y | 239(33.8) | 29(15.5) | 268(29.9) | <0.001 |
| 45-60y | 234(33.1) | 58(31.0) | 292(32.6) |  |
| >60y | 235(33.2) | 100(53.5) | 335(37.5) |  |
| Gender, women (n,%) | 367 (51.8) | 105 (56.1) | 472 (52.7) | 0.323 |
| Herdsman (n, %) | 269(38.0) | 107(57.2) | 376(42.0) | <0.001 |
| Education levels (n, %) |  |  |  |  |
| Primary and lower | 387 (54.7) | 133(71.1) | 520(58.1) | <0.001 |
| Junior high | 170(24.0) | 35(18.7) | 205(22.9) |  |
| Senior high and higher | 151(21.3) | 19(10.2) | 170(19.0) |  |
| Ethnicity (n, %) |  |  |  |  |
| Han | 302(42.7) | 78(41.7) | 380(42.5) | 0.337 |
| Kazakh | 107(15.1) | 37(19.8) | 144(16.1) |  |
| Tajik | 79(11.2) | 23(12.3) | 102(11.4) |  |
| Others | 220(31.1) | 49(26.2) | 269(30.1) |  |
| Number of family members (n, %) |  |  |  |  |
| 1 | 32(4.5) | 11(5.9) | 43(4.8) | 0.723 |
| 2-4 | 455(64.3) | 120(64.2) | 575(64.3) |  |
| ≥5 | 221(31.2) | 56(29.9) | 277(30.9) |  |
| Marital status (n, %) |  |  |  |  |
| Single | 85(12.0) | - | 85(9.5) | <0.001 |
| Married | 569(80.4) | 166(88.8) | 735(82.1) |  |
| Separated | 54(7.6) | 21(11.2) | 75(8.4) |  |
| Family income per member |  |  |  |  |
| <￥500/month | 68 (9.6) | 139(74.3) | 207(23.1) | <0.001 |
| ￥500-1000/month | 84(11.9) | 19(10.2) | 103(11.5) |  |
| ￥1001-3000/month | 427(60.3) | 5(2.7) | 103(48.3) |  |
| >￥3000/month | 129(18.2) | 24(12.8) | 153(17.1) |  |
| Altitude of habitation (m) |  |  |  |  |
| <1000 | 431(60.9) | 120(64.2) | 551(61.6) | 0.199 |
| 1000-3000 | 198(28.0) | 41(21.9) | 239(26.7) |  |
| >3000 | 79(11.1) | 26(13.9) | 105(11.7) |  |
| Current smokers (n, %) | 162(22.9) | 38(20.3) | 200(22.3) | 0.491 |
| Current drinkers (n, %) | 145(20.5) | 39(20.9) | 184(20.6) | 0.919 |
| Body mass index | 27.40±4.29 | 26.80±4.53 | 27.28±4.35 | 0.094 |
| BMI: <23.9kg/m^2^ | 136(19.2) | 41(21.9) | 177(19.8) | 0.608 |
| BMI: 24.0-27.9kg/m^2^ | 279(39.4) | 75(40.1) | 354(39.6) |  |
| BMI: ≥28.0kg/m^2^ | 293(41.4) | 71(38.0) | 364(40.7) |  |
| Abdominal obesity (n, %) | 482(68.1%) | 125(66.8%) | 607(67.8%) | 0.792 |
| CVD (n, %) | 21(3.0) | 2(1.1) | 23(2.6) | 0.195 |
| Diabetes (n, %) | 107(15.1) | 15(8.0) | 122(13.6) | 0.012 |
| Dyslipidemia (n, %) | 139(19.6) | 15(8.0) | 154(17.2) | <0.001 |
| Comorbidity (n, %) | 231(32.6) | 28(15.0) | 259(28.9) | <0.001 |
| Blood pressure(mmHg) |  |  |  |  |
| Systolic blood pressure | 149.46±22.14 | 152.94±14.61 | 150.19±20.83 | 0.042 |
| Diastolic blood pressure | 84.87±13.08 | 89.12±10.70 | 85.76±12.73 | <0.001 |
| CVD, cardiovascular disease. | | | | |
